# Physiochemical characterization of a potential *Klebsiella* phage MKP-1 and analysis of its application in reducing biofilm formation

**DOI:** 10.3389/fmicb.2024.1397447

**Published:** 2024-07-17

**Authors:** Sayani Das, Sandip Kaledhonkar

**Affiliations:** Department of Bioscience and Bioengineering, IIT Bombay, Mumbai, Maharashtra, India

**Keywords:** *Klebsiella pneumoniae*, bacteriophage, phage therapy, biofilm-formation, phage MKP-1

## Abstract

The common intestinal pathogen *Klebsiella pneumoniae (K. pneumoniae)* is one of the leading causes of fatal superbug infections that can resist the effects of commonly prescribed medicines. The uncontrolled use or misuse of antibiotics has increased the prevalence of drug-resistant *K. pneumoniae* strains in the environment. In the quest to search for alternative therapeutics for treating these drug-resistant infections, bacteriophages (bacterial viruses) emerged as potential candidates for in phage therapy against *Klebsiella*. The effective formulation of phage therapy against drug-resistant *Klebsiella* infections demands thorough characterization and screening of many bacteriophages. To contribute effectively to the formulation of successful phage therapy against superbug infections by *K. pneumoniae*, this study includes the isolation and characterization of a novel lytic bacteriophage MKP-1 to consider its potential to be used as therapeutics in treating drug-resistant *Klebsiella* infections. Morphologically, having a capsid attached to a long non-contractile tail, it was found to be a siphovirus that belongs to the class *Caudoviricetes* and showed infectivity against different strains of the target host bacterium. Comparatively, this double-stranded DNA phage has a large burst size and is quite stable in various physiological conditions. More interestingly, it has the potential to degrade the tough biofilms formed by *K. pneumoniae* (*Klebsiella pneumoniae* subsp. *pneumoniae* (Schroeter) Trevisan [ATCC 15380]) significantly. Thus, the following study would contribute effectively to considering phage MKP-1 as a potential candidate for phage therapy against *Klebsiella* infection.

## 1 Introduction

*Klebsiella pneumoniae,* the common intestinal bacterium, belongs to a gram-negative, encapsulated group of bacterium species that causes various infections and is often responsible for causing pneumonia, urinary tract infections, meningitis, and liver infections (Cotton et al., 2000; Paczosa and Mecsas, 2016). Over the last few years, *Klebsiella* has been found to cause most nosocomial infections with an upsurge in mortality rate (Herridge et al., 2020), as the drug-resistance rate in *Klebsiella* has increased to nearly 70% globally (Li et al., 2022). Notably, in contrast to other gram-negative bacteria, this bacterium has a rare ability to cause primary pneumonia in patients (Choby et al., 2020). In humans, this bacterium usually colonizes the mucosal surfaces of the throat region and gastrointestinal (GI) tract. Once introduce into the body, it can display high virulence and antibiotic resistance (Ashurst and Dawson, 2023). The presence of capsular polysaccharides (CPS) surrounding the cell wall is the prime factor that ensures the antibiotic resistance and survival of *K. pneumoniae* in stress conditions. The cellular capsule surrounding the cell membrane of the bacterium play a pivotal role in the evasion of the host defense and serve as a barrier that protects the bacterial cells against antibiotic penetration (Anderl et al., 2000). According to the World Health Organization (WHO), the emergence of resistance in *K. pneumoniae* against salvage therapy (carbapenem antibiotics) is very prevalent nowadays and is noticeable worldwide (WHO, 2023). Consequently, *K. pneumoniae* has been placed on the priority pathogen list with a critical priority that must be taken care of immediately (WHO, 2023).

The formation of biofilms is one of the important aspects of antimicrobial resistance in some specific bacterial species (Uruén et al., 2020). Biofilms are tough film-like structures that are formed due to complex chemical interactions between bacterial species. These are generally composed of one or more bacterial species encased in an extracellular matrix which is composed mainly of proteins, carbohydrates, and genetic materials (DNA and RNA). Reports have suggested that bacteria, when they form biofilms, are protected from host immune responses and antibiotics (Souza et al., 2022). Studies have reported that in most cases, in patients, *K. pneumoniae* forms complex biofilms with other bacterial species such as *Pseudomonas aeruginosa* and *Pseudomonas protegens* during nosocomial infections (Kolenbrander et al., 2010; Joshi et al., 2021).

Due to the reduced effectiveness of antimicrobials to treat drug-resistant *Klebsiella-*associated infections, bacteriophages (bacterial viruses) against *Klebsiella* received high attention as an alternative treatment agents (Herridge et al., 2020). Being the most abundant entities on the earth (Das and Ghosh, 2017), these small viruses are beneficial for humans in many aspects. Their use is not only restrained to treating the bacterial infection as a part of phage therapy but they also could take part in routing out the spread of epidemics caused by the respective host bacterium (Chattopadhyay et al., 1993). The capability to replicate their DNA to form structural protein components inside the host body using the host’s enzymes within a very short time makes phages the potential candidates for fabricating nano-containers to be used as a delivery system (Das and Ghosh, 2017). To date, various literature studies (Anand et al., 2020; Cano et al., 2020; Assafiri et al., 2021; Kumar et al., 2023) have reported the successful use of phages as therapeutics against *Klebsiella* infections. However, the complexity of phage–host interactions remains to be explored further (Chadha et al., 2017), to comment on detail infection strategies. In addition, some studies have suggested that the phages that are isolated in one place likely will not show infectivity against the host bacteria in different places due to the high diversity of the genome and the ever-evolving defense mechanisms of the Enterobacteriaceae group of bacteria (Gencay et al., 2019). Moreover, the host-reliance nature of viruses and bacteriophages frequently encourages the selection for strong compatibility of viruses with potential hosts for a successful infection. Though studies have reported strong correlation between codon usage in viral and host genomes, the selective factors by which this host–virus compatibility evolves remain a matter of presumption (Kula et al., 2018). As an add-on, bacteria often show resistance against respective bacteriophages following antiphage defense mechanisms due to evolution (Hasan and Ahn, 2022). This antiphage system of bacteria usually targets the phage life cycle, including adsorption to the host cell, ejection of DNA into the host, synthesis, the assembly of phage particles, and the release of progeny phages (Hasan and Ahn, 2022). These factors are the major hindrances to formulating effective phage therapy. Thus, there is a continuous search for new phages focusing on different serovars with high lytic ability for practical application (Kropinski et al., 2009). However, to meet the requirements of a potential candidate, a phage, along with the lytic activity, should preferably have a broad host range, large burst size, and high physical stability. In addition, its genome should be thoroughly characterized to look for the presence of any toxin genes (Mallick et al., 2021) that could affect the therapeutic purpose.

In the following study, we have reported the isolation and thorough characterization of a novel phage MKP-1, which is isolated from a sewage sample against the *Klebsiella* strain. This phage was isolated from a water body (Mithi River, Mumbai) where most hospital wastes are dumped (RenukaShinde, n.d.). Therefore, it is more likely to be infectious against clinical strains of *Klebsiella*. The phage showed high lytic activity against different strains of the host bacterium and was stable under varied environmental conditions. It was then thoroughly characterized based on its biophysical, morphological, and genomic characteristics, which further showed anti-biofilm activities. We have tried to perceive the optimum time of incubation of phages with the biofilms for effective degradation of tough host biofilms, the key agent behind antimicrobial resistance.

## 2 Materials and methods

### 2.1 The bacterial hosts

In this study, *Klebsiella pneumoniae* strain MTCC 618 was used as the host organism to isolate lytic phage from the sewage sample. It is a reference strain received from the National Collection of Type Cultures (NCTC 11228), UK, and is authorized by Schroeter (1886) and Abel (1893). The bacteria were cultured in Luria–Bertani (LB) broth and Luria–Bertani agar (LA) medium at 37°C inside a shaker incubator following all the standard biosafety guidelines. The host bacteria were grown till log phage (0.6–0.8 OD600) before performing all the phage-based assays.

### 2.2 Isolation, purification, and preparation of high-titer phage sample

For isolating phages against the host *Klebsiella*, the water sample was collected from the Mithi River which flows through the Mumbai suburban and is one of the most polluted rivers in Mumbai that possesses hospital wastes. The sample was processed further to check the presence of bacteriophage against the host following protocols as discussed in Adams (1963). In brief, 25 mL of the water sample was taken and centrifuged, and the supernatant was mixed with 5 mL of log-phase host bacterial culture, in which 25 mL of phage broth medium (Himedia, M936) was added for nourishment. The mixture was then incubated for 24 h at 37°C in a shaker incubator which rotates at 150 rpm. After incubation, the mixture was centrifuged at 10,000 rpm for 10 min at 4°C, the supernatant was filtered through a 0.22 μm membrane filter, and spot assay was conducted using the top-agar overlay method. After observing a clear spot-on bacterial lawn, a plaque was taken and, subsequently, cultured for multiple generations to obtain homogeneous plaques. The phage sample was further concentrated by ultracentrifugation for 2 h and 30 min at 25,000 rpm at 4°C using the Type 70Ti fixed-angle rotor and Beckman Coulter ultracentrifugation. After concentrating, the sample was then purified via sucrose step-gradient ultracentrifugation for 2 h at 25,000 at 4°C using the SW41 swinging rotor, followed by dialysis against the SM buffer containing 1 M NaCl.

### 2.3 Determination of optimum MOI by killing curve assay

To check the lytic activity of this phage against the host and determine the optimum multiplicity of infection (MOI) of this phage, the killing curve assay was performed. For instance, freshly grown host (log phase) culture was added in multiple wells of the 96-well plate and infected with phages at different MOIs of 0.1, 1, and 10. LB broth and uninfected host culture were used as blank and control, respectively, followed by an evaluation of the optical density of the host cell by the optical reader MultiscanGo (Thermo Fisher Scientific) at 600 nm (OD600).

### 2.4 Host range analysis

To check the lytic module of the isolated phage, the host range was determined using two other strains of *Klebsiella pneumoniae* (MCC 4407 and MCC 2716) and other gram-negative bacteria such as *Escherichia coli*, *Salmonella typhi*, and *Pseudomonas aeruginosa*. The two *Klebsiella* strains MCC 4407 and 2,716 are environmental strains that were isolated from rhizospheric soil sample. To check the infectivity of phage MKP-1, approximately 100 μL of bacteria from the overnight culture was mixed with 0.8% soft agar and poured onto a Luria–Bertani agar (LA) plate, and a spot assay was performed by adding 10 μL of the phage lysate to the bacterial lawn.

### 2.5 Morphological studies by transmission electron microscopy

The phage morphology was studied by employing negative stain and electron microscopy analysis. In brief, carbon-coated copper grids were glow-discharged, and 3 μL of the purified phage sample was drop-casted onto them. The sample was then negatively stained with 2% uranyl acetate for 30 s, and grids were observed under Themis 300 Transmission Electron Microscope of Thermo Fisher Scientific for visualization.

### 2.6 Biophysical characterization

#### 2.6.1 One-step growth curve and adsorption kinetics

The one-step growth experiment was performed to assess the growth of the phage inside the host cell. For the experiment, log phase host culture having a concentration of 10^8^ cells/ml was infected with the phage at a multiplicity of infection (MOI) of 0.1. The host–phage mixture was incubated at 37°C for 5 min inside a shaker incubator. The phage-bacterial mixture was then centrifuged, the supernatant was taken out, and the pellet was re-suspended in 20 mL of fresh LB broth and incubated at 37°C. During incubation, aliquots were taken every 5 min for 120 min and assayed for the presence of total phage by the soft agar overlay method. The growth curve was plotted by calculating the number of phages (pfu/ml) present concerning time. From the curve, burst size was calculated as the ratio of the final count of liberated phage particles to the initial count of the infected host bacterium cells during the latent period.

For studying the adsorption kinetics, the bacterial culture was grown till the exponential phase and was infected with the phage at a multiplicity of infection of 0.1. This bacteria-phage mixture was incubated for 40 min at 37°C in a shaker incubator. Approximately 200 μL of the sample was taken recurrently at 2-min interval and centrifuged. The supernatant obtained containing the free phage was assayed by the serial dilution and plating method. The adsorption coefficient for this phage was calculated as described for bacteriophage by T4 by Stent (1963).

#### 2.6.2 Sustainability studies on this phage under temperature, pH, salinity, and UV stress

##### 2.6.2.1 Thermal stability assay

To be included in the regime of phage therapy, a phage needs to be stable in a broader environmental condition. Therefore, to test the stability of this phage under various temperatures, approximately 200 μL of phage aliquots (10^12^ pfu/ml) was incubated under various temperature ranges (10°C, 20°C, 30°C, 40°C, 50°C, 60°C, 70°C, and 80°C) for 1 h, and survivability of the phage under heat stress was analyzed by the phage titer assay following serial dilution and the soft agar overlay method.

##### 2.6.2.2 pH stability assay

When phages are given to the human body as part of phage therapy, they are exposed to various environments of different pH. Therefore, their susceptibility toward different pH would contribute to deciphering the appropriate route of administration of phages. To analyze the phage susceptibility toward different pH environments, phage dilutions were prepared from concentrated and purified phage stock using SM buffer of varying pH ranges (pH 2–14). The phage dilutions were then kept at room temperature at least for 1 h. The dilutions were kept at room temperature (RT) for 1 h before assaying their survivability by serial dilution and soft agar overlay plaque assay.

##### 2.6.2.3 Sustainability studies under UV light

Ultraviolet radiation (UV) is extensively used for inactivating microorganisms. Bacteriophages or phages show no more exceptions and are also highly susceptible to UV radiation. However, in some cases, it has been observed that phages are more stable under stress conditions in comparison with their host bacterium. Therefore, to study its sustainability under UV exposure, phage aliquot having a concentration of approximately 10^9^pfu/ml was exposed as a thin layer (1 mm thick) in a plastic Petri dish and kept at a distance of 50 cm under an 18-W Philips germicidal lamp that primarily emits 254-nm UV light. Aliquots of phage were removed every 5 s and assayed for surviving phages by plaque assay.

##### 2.6.2.4 Salinity tolerance studies

The salinity of buffers also influences the growth of microbes and phages. Phages to be used in phage therapy should be tolerant to saline conditions as they might be exposed to salinity stress. Therefore, the tolerance to salinity of this phage was tested according to a previously described method with little modification (Giantommaso et al., 2018). In brief, phage dilutions were prepared from concentrated stock using SM buffer with varying salt concentrations (100–600 mM) and kept at room temperature for 4 h, and the viability of the phages was checked by plaque assay.

### 2.7 Phage–host interaction studies employing transmission electron microscopy

#### 2.7.1 Negative staining and electron microscopy on phage–host interaction

For an effective infection, phages should recognize and interact with the host cell receptor by chemical cross-talk. Tailed phages generally initiate infection by interacting with the host cell receptors through their tails. We wanted to evaluate the structural basis of the host–pathogen interaction by electron microscopy. In brief, the host cells were incubated with the phage for different time points (5, 10, 15, 20, 25, 30, 35, and 40 min) at an MOI of 10. After infection, the phage-bacterial mixture was centrifuged, and the pellet was resuspended in 20 μL of LB broth. From that concentrated sample, approximately 5 μL of the sample was drop-casted onto the carbon-coated copper grids, washed with buffer, and negatively stained with 2% uranyl acetate to image under a 300 kV Transmission Electron Microscope.

#### 2.7.2 Ultra-thin sectioning and electron microscopy on phage-infected host cells

To study the bacteriophage–host interaction at the cellular level, ultrathin sectioning and electron microscopic techniques were used. In brief, aliquots of bacteria–phage suspensions were incubated for different periods, i.e., 5, 15, and 40 min inside a shaker incubator. After infection, the phage-bacterial suspensions were first fixed with 3% buffered glutaraldehyde (prepared in Na-cacodylate buffer) for 4 h at 4°C followed by a second fixation with 1% osmium tetroxide for 1 h. The fixed samples were then dehydrated in a graded series of acetone (30, 50, 70, 90, and 100%) and embedded in Agar 100 resin (Agar Scientific). Thin sections (40 nm) were cut from the embedded resin blocks in a Leica Ultracut UC7 ultramicrotome, and the sections were double-stained with 2% uranyl acetate and 0.2% lead citrate. The ultrathin sections were then observed under the transmission electron microscope.

### 2.8 Host biofilm formation and effect of the phage on the bacterial biofilms

To test the biofilm degradation activity of this newly isolated phage, bacterial biofilm was grown on a glass coverslip and 96-well plates. The films formed by the host were then treated with the phages and analyzed by scanning electron microscopy and by measuring optical density using plate reader, respectively.

#### 2.8.1 Biofilm formation on glass coverslips and phage treatment

To visualize phage efficacy in reducing host bacterial biofilms, host bacteria were allowed to form biofilms onto a glass coverslip. Two sets of experiments were set up, where in one set, biofilms were formed by the host bacteria *Klebsiella pneumoniae* only, and in another set of experiments, biofilms were formed by a mixed infection of *Klebsiella pneumoniae* and *Pseudomonas aeruginosa,* which mimics the clinical aspects of biofilm formation in the patient’s body (Kolenbrander et al., 2010; Joshi et al., 2021). In short, 100 μL of an overnight bacterial culture was dispensed onto the glass coverslips (22 mm × 22 mm) which were kept inside a Petri dish. The Petri dishes containing the coverslips were then kept at 37°C and incubated for 48 h to allow the bacteria to form the biofilms. Following incubation, some coverslips with biofilms were treated with PBS buffer, and others were treated with the phage for different periods (6 h, 12 h, 18 h, and 24 h) while incubating at 37°C.

#### 2.8.2 Scanning electron microscopy (SEM) on the bacterial biofilms

The bacterial biofilms of both control and phage-treated samples formed on glass coverslips were prepared for scanning electron microscopic (SEM) analysis as described by Jahid et al. (2013), with some modifications. Precisely, biofilms were fixed with 3% buffered-glutaraldehyde (EMS, United States) and prepared in 0.1 M Na-Cacodylate buffer at 4°C overnight. Following fixation, samples were serially dehydrated with 30, 50, 70, and 90% ethanol with 10-min incubation for each and finally with 100% ethanol that was used twice for 15 min each. The dehydrated biofilms were then air-dried, sputter-coated with platinum, and visualized under a JEOL-7600F Field Emission Gun-Scanning Electron Microscope (Japan).

#### 2.8.3 Optical quantification of biofilm upon phage treatment

To validate the efficacy of MKP-1 as an anti-biofilm agent, host biofilms were developed in 96-well plates following the methods described by Coffey and Anderson (2014), with some modifications. In brief, host bacteria were grown overnight at 37°C, and upon diluting to 1:10 in LB medium, 200 μL of the diluted culture was added to multiple wells of two 96-well plates. The plates were then incubated at 37°C for 24 h and 48 h separately. After respective incubation, extra culture was removed by inverting the plates and washed with 1X PBS buffer to remove the free cells, and the phage sample was added at different MOIs (1 and 10). Upon incubation at 37°C for 24 h, the wells were washed with 1X PBS, air dried, and stained with 0.1% crystal violet for 30 min. Upon removing the extra stain from the wells, the optical densities of the control and phage-treated biofilm were measured by a microplate reader at 595 nm (Thermo Scientific MultiscanGo).

### 2.9 Genomic analysis of the phage MKP-1

The phage DNA was extracted from high-titer (10^14^ pfu/ml) phage stock using a DNA isolation kit (QIAGEN, Germany), according to the user’s manual provided with the kit. The purity and concentration of the DNA were checked through 1.0% agarose gel and nano-drop. The purified DNA was further sent for sequencing at the Hi-Gx360 sequencing facility in Mumbai, India. The sequencing effort was carried out using the MiSeq instrument, and contigs were analyzed by the MultiQC modulator tool. The ORFs were predicted by using Pharokka (version 1.4.1) (Bouras et al., 2023) and further validated by the RAST genome annotation web server (McNair et al., 2018). The presence of t-RNA was scanned through tRNAscan-SE (Lowe and Eddy, 1997). The lifecycle of the phage was confirmed by PHACTS (McNair et al., 2012) software packages. The phage genome was aligned with the genome of other Siphoviridae *Klebsiella* phages using Mauve software packages (Darling et al., 2004). Based on the whole genome of the phage, the phylogenetic tree was constructed using ViPTree (Nishimura et al., 2017), and neighbor-joining phylogenetic trees were constructed based on endolysin protein with the default pipeline “ONE CLICK” at Phylogeny. fr (Dereeper et al., 2008).

### 2.10 Statistical analysis

All the statistical analyses were performed using Origin software packages (2022). All the graphical analyses were performed with *N* = 3/triplicated data sets, and data are represented as mean ± SD values. For biofilm studies, we performed Student’s t-test, and the significance was analyzed by *p*-value (less than 0.05).

## 3 Results

### 3.1 Isolation, purification, and analysis of the lytic activity of the phage

**Figure 1:**
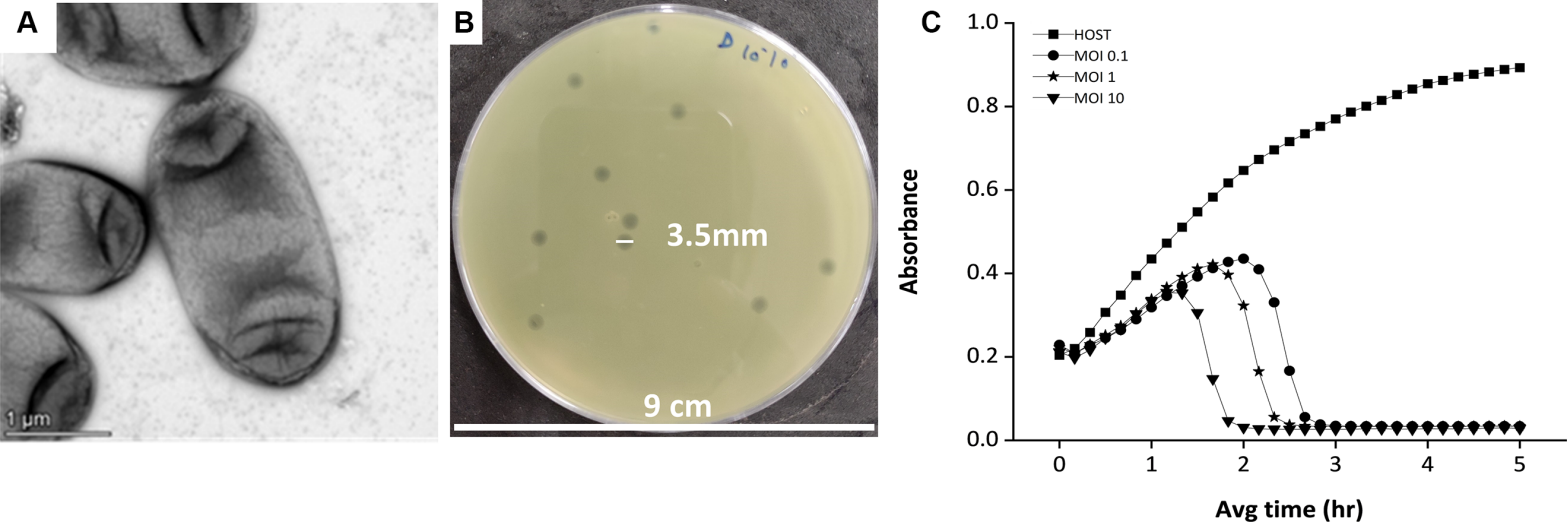
The host and the lytic activity of the phage against the host. **(A)** Electron micrograph of *Klebsiella pneumonia* strain 618 which was used as a host organism for the study. The image shows the presence of a capsule surrounding the bacterium cell. **(B)** The phage MKP-1 forms clear, homogeneous plaques on the host bacterium lawn. **(C)** Killing curve of phage MKP-1 at MOI of 0.1,1 and 10. Phage treatment against *Klebsiella pneumoniae* showed a steady decrease in the host’s cell density (OD at 600 nm), concerning time in comparison with the control host sample.

The phage was isolated against the *Klebsiella pneumoniae* bacteria, which is an encapsulated, gram-negative, non-motile bacterial species (Figure 1A). While infecting the *Klebsiella* strain, the bacteriophage has shown clear, big, round plaque on the bacterial lawn which is approximately 3.5 mm in diameter (Figure 1B). The plaques were double-layered as halos were visible surrounding the clear zone, indicating the secretion of enzyme depolymerase (Sanmukh et al., 2023). The depolymerase activity of the phages often confers high lytic activity and potential biofilm-degrading ability of phages (Gutiérrez et al., 2015; Guo et al., 2017; Sanmukh et al., 2023). The killing curve assay, where the host bacterium was incubated with the phage for 5 h, showed a gradual decrease in the host concentration upon phage infection (Figure 1C) in an MOI-dependent manner. The MOI 10 was found to be optimum for this phage infection, as at this high MOI, the phage was capable of reducing the host cell density toward blank within 2 h of infection.

### 3.2 Host range

The host range of phage MKP-1 manifested its lytic activity toward multiple strains within the target bacterium species. Along with the host bacterium *Klebsiella pneumoniae* strain ATCC 618, the phage showed infectivity against two other *Klebsiella* strains (MCC 4407 and MCC 2716). Thus, we could infer that it is a polyvalent phage that showed infectivity against multiple strains of *Klebsiella*. However, when its infectivity was tested against other common gram-negative bacterial species, it showed no lytic activity against them, and the results are presented in Table 1. Thus, the phage showed a broader host range within the same target species but did not show any cross-infectivity by infecting different bacterial species.

**Table 1:** Host range spectrum of phage MKP-1.

Organism	Strain used	Phage susceptibility
*Klebsiella pneumoniae*	MCC 618	(++)
	MCC 4407	(++)
	MCC 2716	(++)
*E. coli*	DH5 alpha	(−−)
*Salmonella typhi*	Ty2	(−−)
*Pseudomonas aeruginosa*	MTCC 424	(−−)

The infectivity of phage MKP-1 was tested against different strains of *Klebsiella* and other gram-negative bacterial species, and it showed host specificity by infecting different *Klebsiella* strains but no other bacterial species.

### 3.3 Morphology of the phage MKP-1

**Figure 2:**
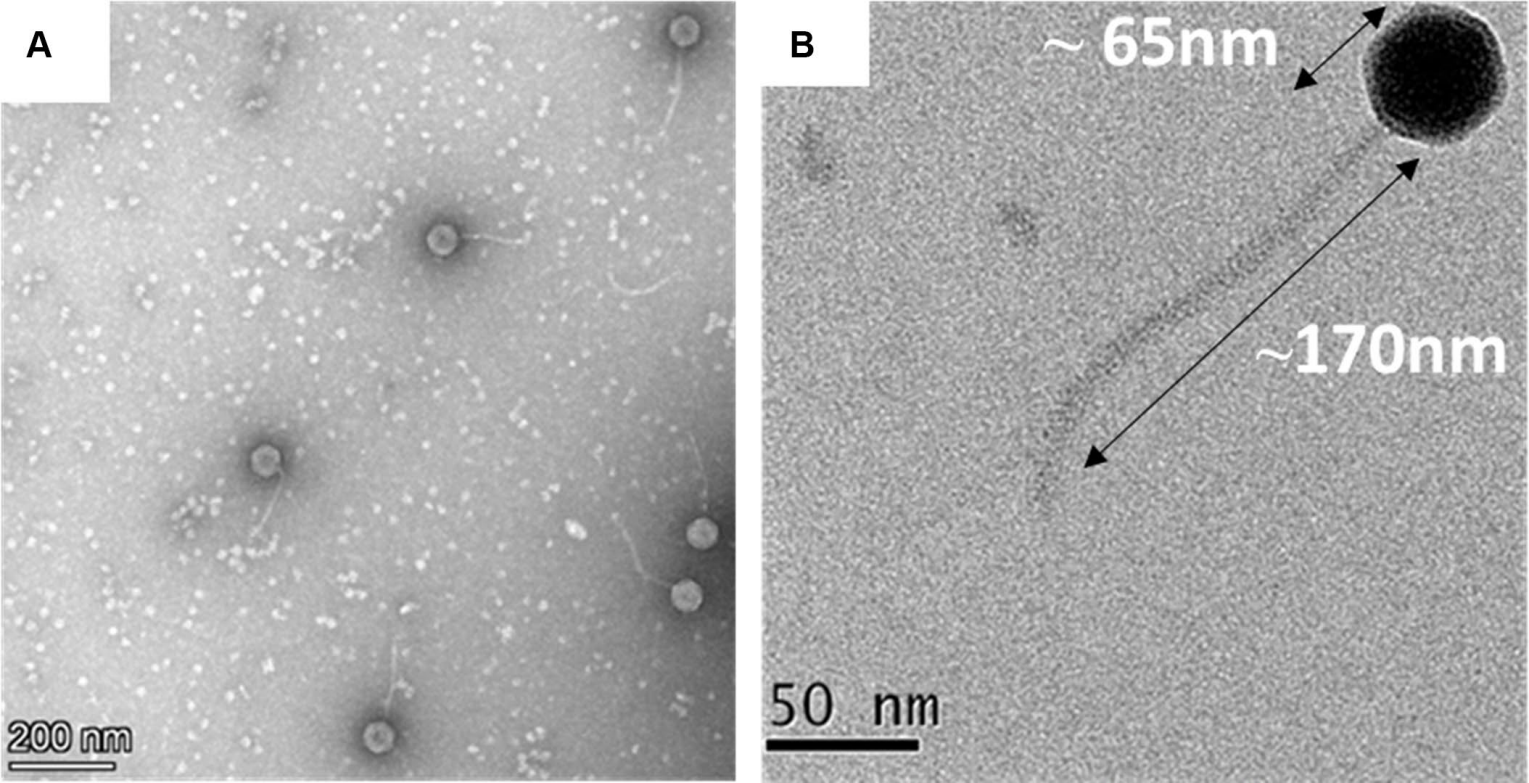
Morphology of the phage MKP-1. **(A)** Negative stained image of the phage MKP-1 showing a hexagonal head attached to a long non-contractile tail. **(B)** The magnified image of the phage shows that the capsid is comparatively small and is approximately 65 nm in diameter. It is attached with a long tail of approximately 170 nm in length.

The transmission electron microscopic analysis of the negatively stained MKP-1 phage has revealed its detailed structural outlines. It has a hexagonal head of ~65 nm in length and ~ 55 nm in width (*n* = 10) (Supplementary Figure S1), which is attached to a long non-contractile tail of length ~ 170 nm (*n* = 10) and breath of ~5 nm (Figures 2A,B). The head is attached to the tail through a head–tail connector protein which is approximately 8 nm in diameter. According to the updated classification of the International Committee on Taxonomy of Viruses (ICTV), being a tailed phage with icosahedral capsid, phage MKP-1 belongs to the class Caudoviricetes, which generally infects archaea or bacteria. In addition, having a non-contractile tail, it could be called a ‘siphovirus’ according to the updated ICTV list.

### 3.4 One-step growth curve and adsorption kinetics of the phage MKP-1

**Figure 3:**
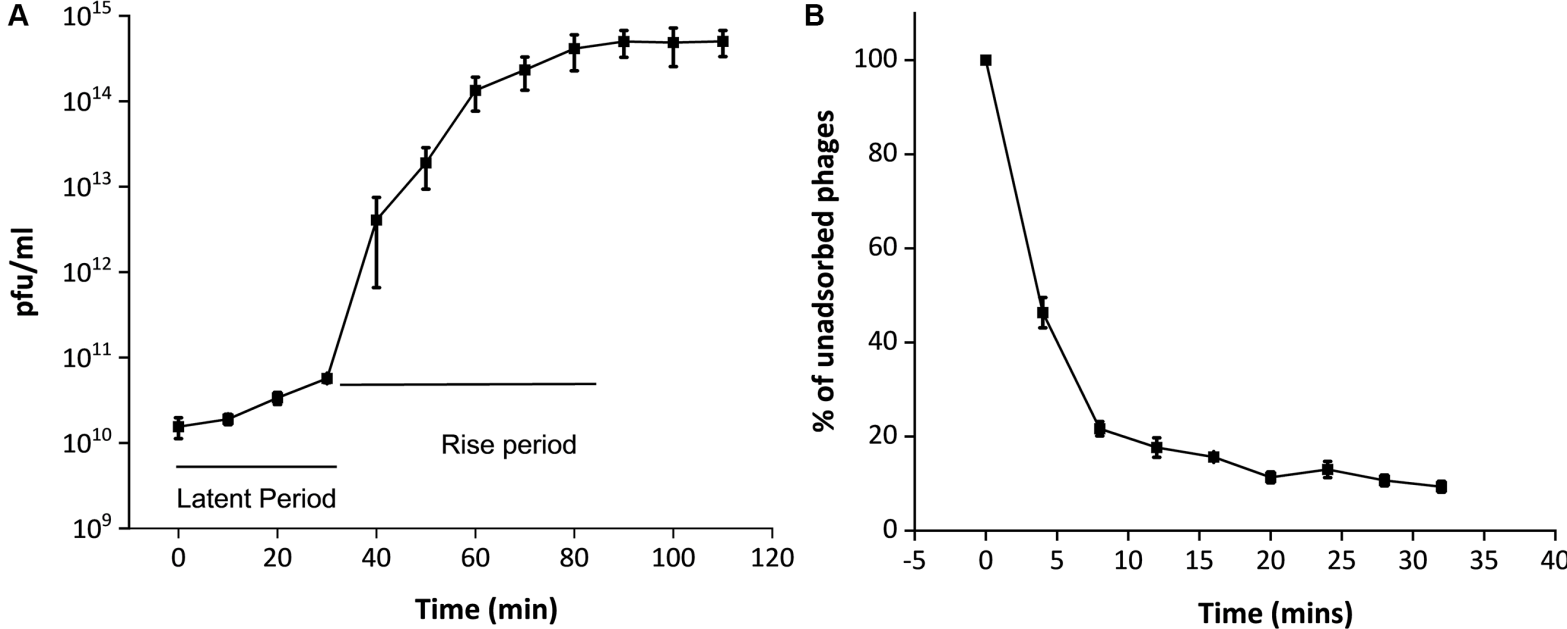
One-step growth curve and adsorption kinetics of phage MKP-1. **(A)** The one-step growth curve showed that this phage MKP-1 has a long latent period of approximately 40 min and a rise period of approximately 50 min. **(B)** The adsorption kinetics also justify the growth curve as it shows approximately 20% of the total phage is still left unabsorbed after 20 min of infection with the host.

Upon infecting the host bacterium, the growth curve of the phage MKP-1 is presented in Figure 3A. This shows that phage MKP-1 has a long latent period of approximately 40 min when the phage transfers its genome to the host cell and prepares necessary proteins before bursting out of the host cell. The latent period is followed by a rise period of 50 min when there is a sharp increase in phage concentration after which the growth ceases. The burst size (the number of phage particles per infected bacterium) was calculated to be approximately 260, which can be considered as a large value relating to the burst size.

The adsorption kinetics assay (Figure 3B) showed that the adsorption rate of this phage was quite high for the initial 5 min, which then started decreasing with time. Considering the presence of free phages, the adsorption constant was calculated to be approximately 1.4 × 10^−4^ mL/min for the initial 5 min of infection.

### 3.5 Stability studies

**Figure 4:**
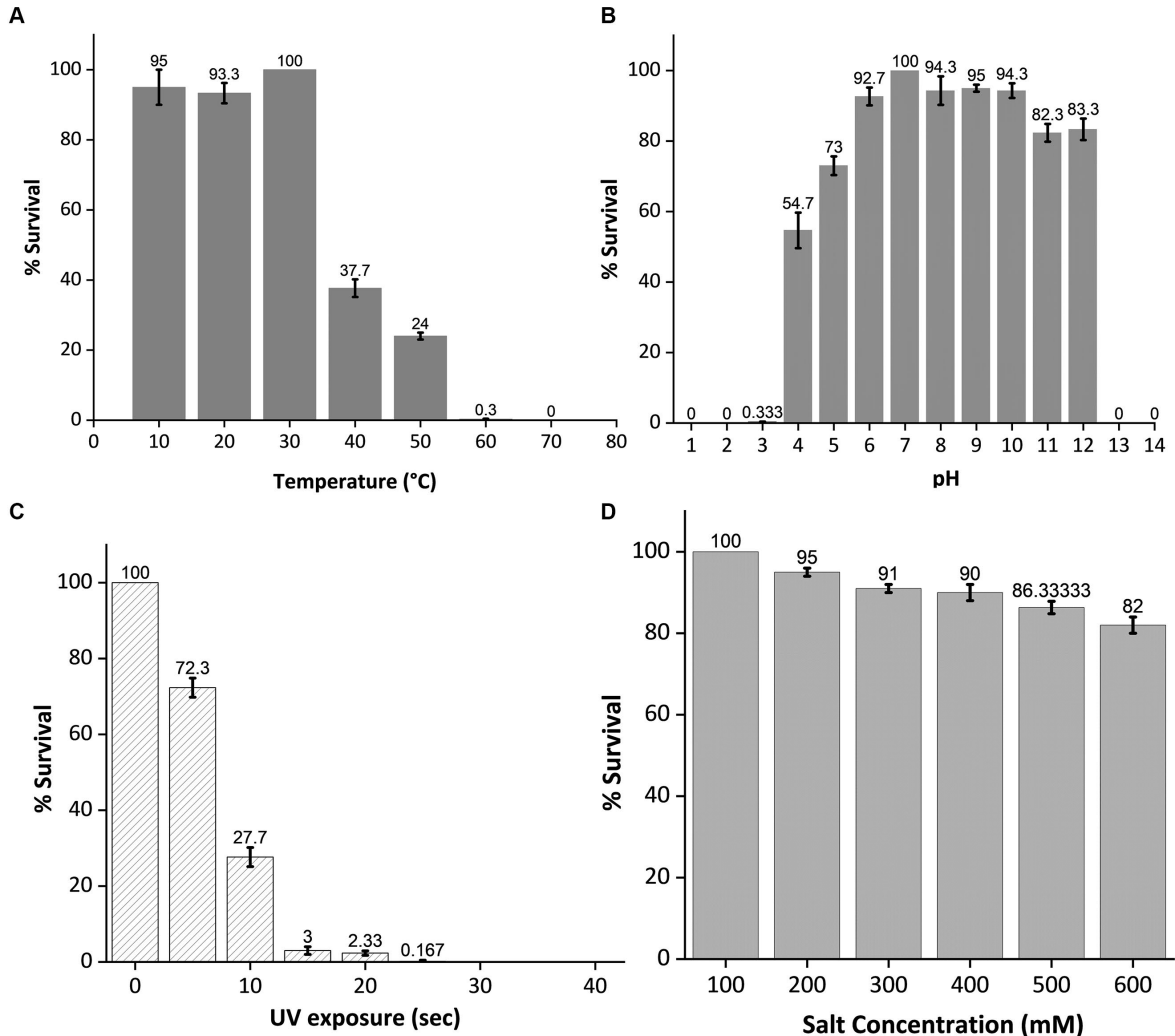
Stability test of phage MKP-1 under temperature, pH, ultraviolet radiation, and salinity stress. All the data were normalized considering control as 100%. **(A)** The temperature stability curve of the phage at different temperatures showed its thermostable nature as phage colonies were observed at 60°C. **(B)** pH inactivation curve showing its stability under a broad range of pH values. This phage showed high stability in both acidic and basic conditions. **(C)** The inactivation curve of phage MKP-1 under UV exposure showed its susceptible nature toward UV exposure as more than 50% of the phage is getting inactivated within 10 s of UV exposure. **(D)** The stability curve of phage MKP-1 under saline conditions shows that it is stable in the high range of salinity.

To assess its stability under various environmental stress factors, phage MKP-1 was propagated under a broad range of temperatures, pH, salinity, and UV exposure. The stability of this phage under various temperatures and pH ranges is presented in Figures 4A,B, which showed its high stability under a wide range of temperature (10°–60°C) and pH (3–12). Approximately 25% of the phage was still active at 50°C. While performing the pH stability assay of this phage, it is observed that acid has more inactivating effects than bases as nearly 80% of the phage survived in the pH range of 11–12, whereas the percentage of survival decreases nearly to 0.33% at pH 3. The phage stability against ultraviolet (UV) radiation is shown in Figure 4C, which confirms its high susceptibility against UV. Merely 5 s of UV exposure could decrease the phage survivability to 70%, and within 20 s, the survivability decreased 2%. Furthermore, when phage susceptibility was tested against salinity (Figure 4D), it was observed that the phage was quite resistant to the salinity as nearly 80% of the phage population still survived at 600 mM NaCl buffer, which was similar to the salinity of seawater.

### 3.6 Phage–host interaction study by transmission electron microscopy

#### 3.6.1 Transmission electron microscopy on host–phage interaction

**Figure 5:**
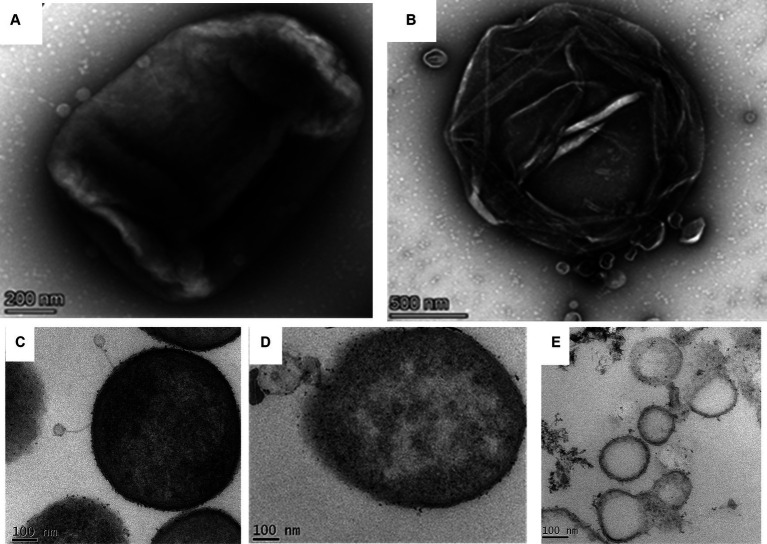
Phage bacterial interaction study by transmission electron microscopy. **(A)** Negatively stained image of the phage interacting with the host. Intact phages are observed to be attached to the host while interacting with the host through their tails. **(B)** Infected host cell after 40 min of incubation with the phage. The image shows completely lysed bacteria due to phage lysis. **(C)** The ultrathin-sectioning image of the phage-infected bacterial cells after 5 min of incubation. Intact phages are attached to the host cells. **(D)** Thin-sectioning image of infected bacteria after 20 min of incubation. Image showing disruption of the bacterial membrane and leaking of cell constituents. **(E)** Thin-sectioning image of phage-infected bacteria after 40 min of infection showing complete lysis of host cells and leakage of cellular material.

The negatively stained phage-infected bacteria showed the presence of intact phages attached to the host bacterium after 5 min of infection (Figure 5A). The image also presented the undamaged cell membrane surrounding the host cells. On the contrary, when the host was incubated with the phage for 40 min, it showed complete lysis (Figure 5B). The cell wall was damaged completely, and subsequently, the cellular material came out from the cell.

#### 3.6.2 Ultrathin sectioning of the phage-infected host bacterium at different incubation times

The micrographs containing ultrathin sections of the host *K. pneumoniae* cells, infected with the phage MKP-1 for different time points of 5 min, 15 min, and 40 min, are shown in Figures 5C–E, respectively. After 5 min of infection with the phages, the negatively stained thin-section image of the host cell showed intact phages attached to the bacteria and are yet to release their DNA (Figure 5C). When the host cells were infected with the phages for 15 min, the disintegration of membranes of the host cell was observed in the micrograph, as the phage might have transferred their DNA to the host upon receptor-mediated binding with the host cell membrane (Figure 5D). However, after approximately 40 min of infection with the phage, the host cells were observed to be completely disintegrated, and the cellular material came out from the cell (Figure 5E).

### 3.7 Biofilm degradation ability of phage MKP-1

The biofilm degradation ability of this phage was determined by the scanning electron microscopic analysis of the control and phage-treated bacterial biofilms formed on the glass coverslips. The findings were further validated by the optical method following crystal violet staining and viable cell count technique.

**Figure 6:**
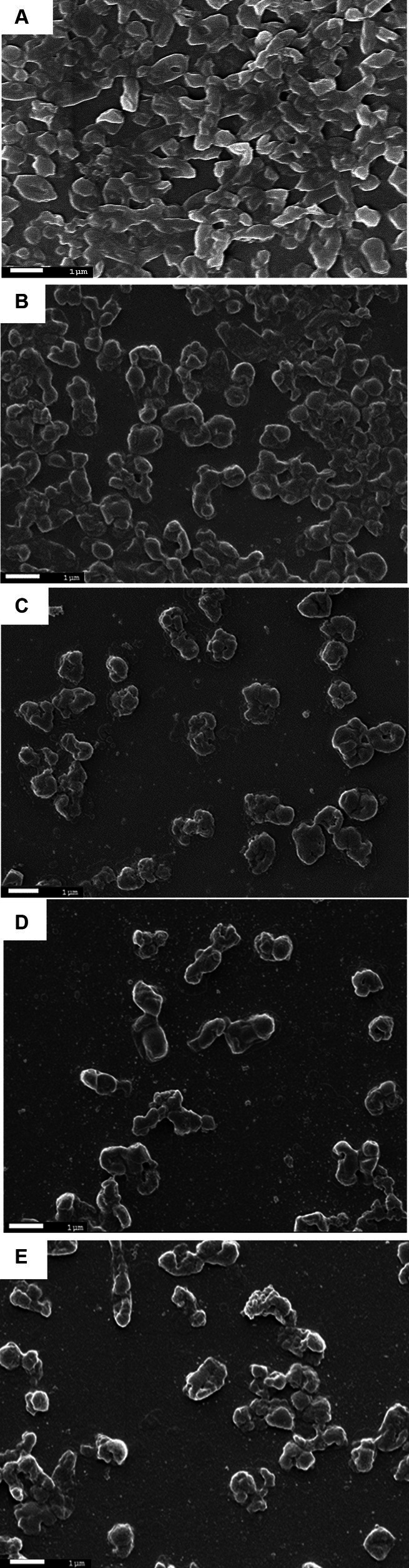
Scanning electron micrographs of biofilms formed by the host organism *Klebsiella pneumoniae*. **(A)** Showing host biofilms formed on a coverslip after incubation of 48 h. **(B)** Showing degraded *Klebsiella* biofilms due to phage treatment for 6 h. **(C)** *Klebsiella* biofilm upon phage treatment for 12 h. **(D)** Degraded host biofilms after 18 h of phage treatment. **(E)** Host biofilms after 24 h of phage treatment.

The SEM analysis of phage-treated bacterial biomass is presented in Figure 6. The disruption of biofilm was observed in phage-treated host biofilm for the control host biofilm (treated with buffer). The micrograph of the uninfected host biofilm is presented in Figure 6A, showing the presence of a continuous, tough film of the host bacterium. However, degradation of the host biofilm was observed upon phage treatment for different time points of 6 h, 12 h, 18 h, and 24 h, respectively (Figures 6B–E). While analyzing the optimal incubation time of host biofilm with the phage for effective degradation of the biofilm, it was observed that phage infection for 12 h exhibits more efficacy in reducing host biomass in comparison to infection for 6 h, 18 h, and 24 h.

**Figure 7:**
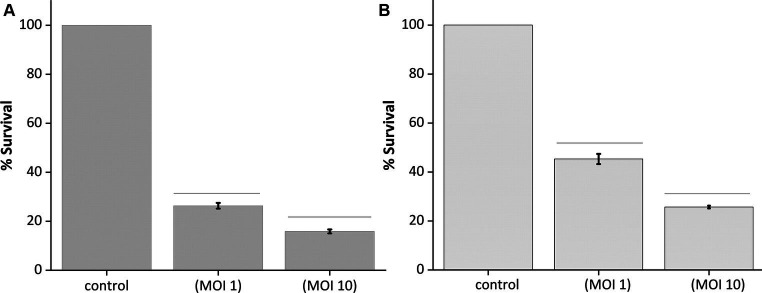
Quantification of biofilm lysis activity of phage MKP-1. **(A)** The graph shows the reduction of 24 h biofilm of host *Klebsiella pneumoniae* due to phage treatment at MOI 1 and 10. **(B)** Showing reduction of 48 h host biofilms due to phage infection at MOI 1 and 10. All the values were tabulated as mean ± SD.

The optical quantification of host biofilm upon phage infection corroborates the previous findings, as the optical density reading at 595 nm of the 24-h and 48-h host biofilms upon phage infection at different MOIs (1 and 10) showed significantly reduced bacterial biomass after 24 h of phage infection (Figures 7A,B). In the case of 24-h biofilm, nearly 70% of the biomass got cleared at MOI 1 and 10; there was a clearance of nearly 80% of the host biomass (Figure 7A). Accordingly, for the 48-h grown biofilm, phage infection at MOI 1 could reduce the host biomass by 50%, and at MOI 10, nearly 70% of the host biomass was reduced (Figure 7B).

The effect of phage infection on the mixed bacterial biofilm formed by the interaction between *Klebsiella pneumoniae* and *Pseudomonas aeruginosa* was also evaluated by quantitative assay based on host cell density and SEM analysis. The findings of the quantitative assay and SEM analysis are presented in Supplementary Figures S3, S4, showing limited degradation of mixed biofilm not as significant as in the case of biofilms by *K. pneumoniae* alone.

### 3.8 Genomic analysis of phage MKP-1

**Figure 8:**
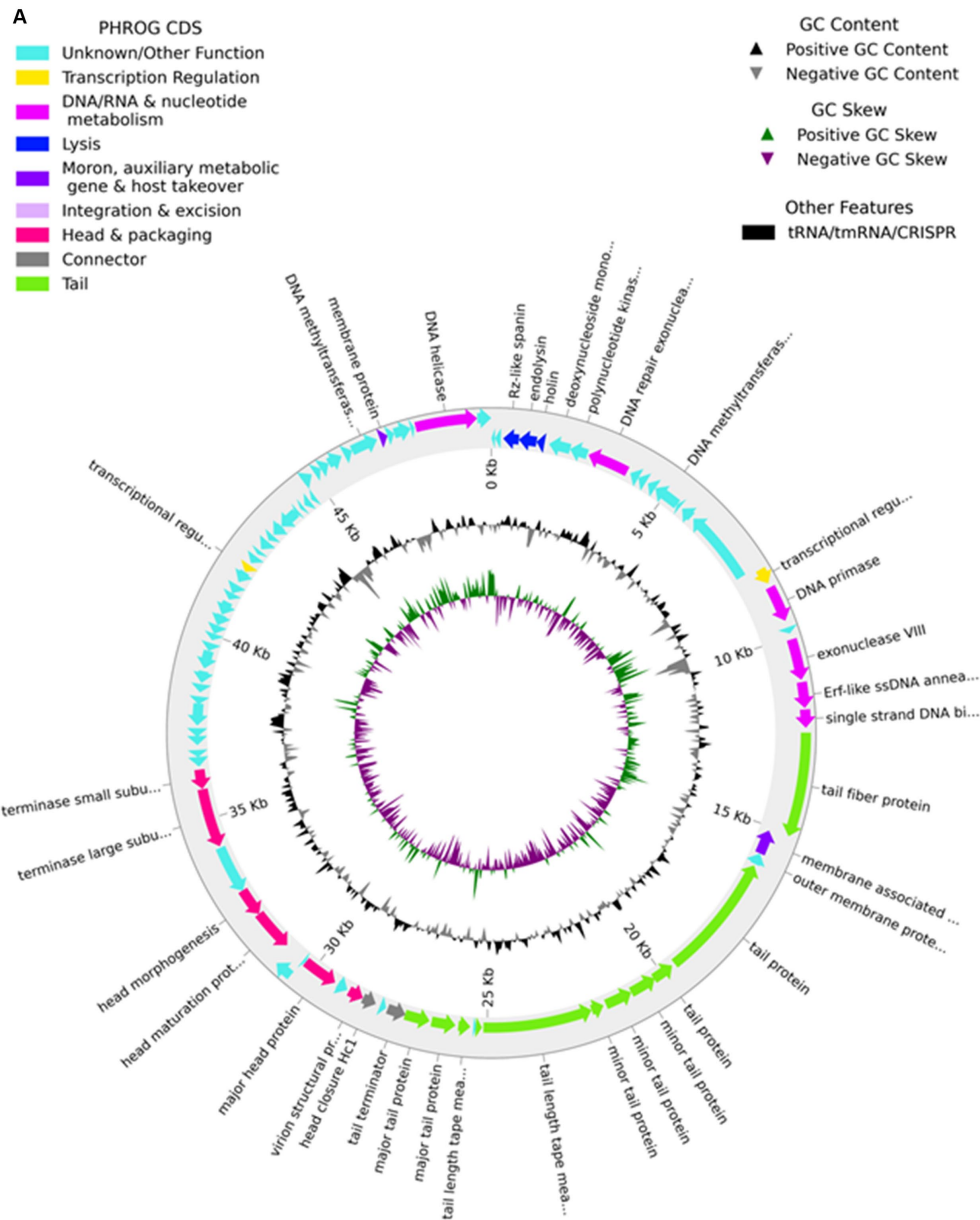

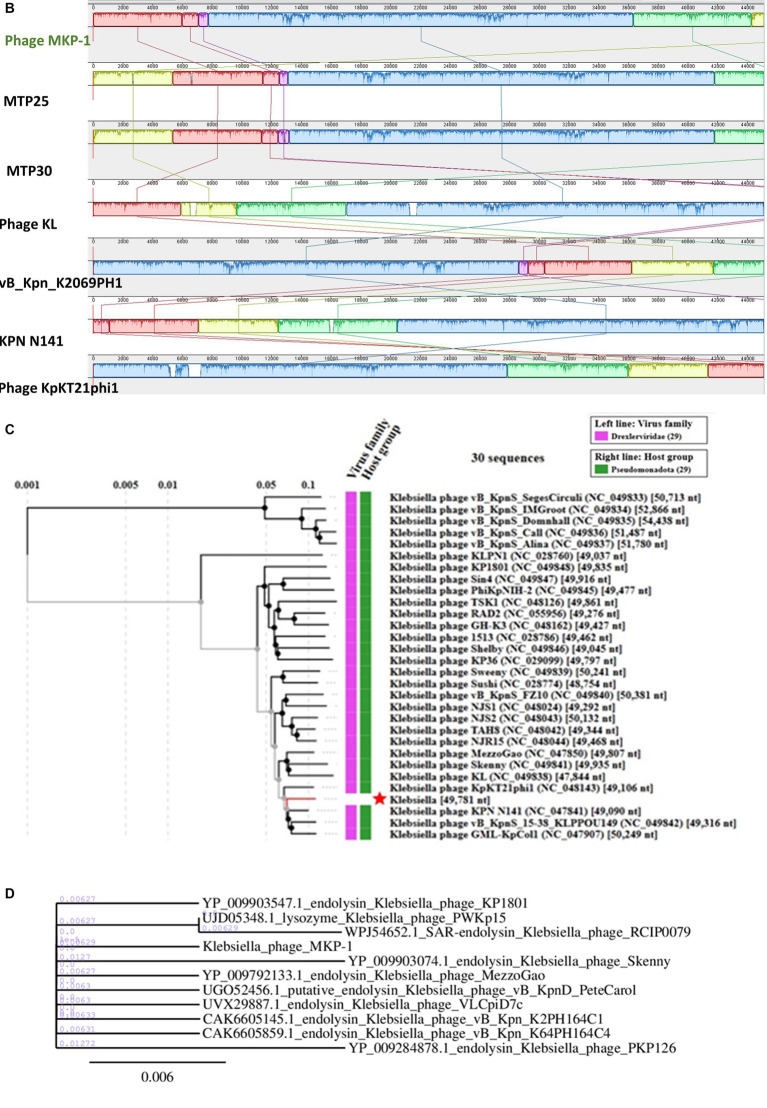
Genomic analysis of phage MKP-1. **(A)** Genomic map of phage MKP-1. **(B)** Schematic representation of multiple sequence alignment of phage MKP-1 DNA with six other closely related *Klebsiella* phages using Mauve. The solid blocks show conserved regions across the phage genome. **(C)** Phylogenetic tree of phage MKP-1 genome with other 30 *Klebsiella* phages generated by VipTree. **(D)** The phylogenetic tree was constructed based on the endolysin protein of the phages.

The genome of phage MKP-1 (Acc. No. PP627509) was isolated, and purity was checked through 1% agarose gel electrophoresis (Supplementary Figure S2). The phage genome is found to consist of 49,781 bp, which possesses a GC content of approximately 51.05%. Thus, the genome of this phage is comparable with other *Klebsiella* phages that have been reported so far (Table 2). The schematic map of the whole genome of phage MKP-1 is shown in Figure 8A, where CDS is marked with different colors based on the prediction. The genes were mostly annotated based on their specific functions, for example, structural genes, genes involved in genome packaging, genes for host cell lysis, and genes for DNA replication and metabolism. Further analysis showed that the genome does not contain any harmful gene and gene for lysogeny. The genome of this phage has shown to have the highest sequence identity (96% identity) with *Klebsiella* phage mtp25 [NCBI Acc. No. OX335391]. The genome analysis of this phage has predicted the presence of approximately 80 open reading frames (ORFs). These 25 ORFs (31%) could be annotated based on their functions. The BLAST analysis of the phage genome showed that taxonomically this phage belongs to the Webevirus family of phages of class *Caudoviricetes*. While scanning the presence of tRNA in the genome, we found that the MKP-1 genome does not contain tRNA-encoding genes, suggesting that it takes over the host’s codon usage pattern during its life cycle.

**Table 2:** Comparative analysis of MKP-1 phage genome.

Phage	Accession number	Genome size (bp)	Identity%
MKP-1	PP627509	49,781	100
mtp25	OX335391.1	50,132	97
Mtp30	OX335411.1	49,926	95
phage vB_Kpn_K2069PH1	OY757093.1	49,869	97
phage vB_KpnS_2811	LR757892.1	49,131	96
phage vB_KpnS-VAC8	MZ428226.1	48,933	95
phage KPN N141	NC_047841.1	49,090	97

The phage genome analysis by NCBI Blast is presented in the table showing percentage similarities with other *Klebsiella* phages.

#### 3.8.1 Protein profile of The phage genome

The phage genome encodes most of the essential proteins that control the phage lifecycle (Supplementary Table S1). The proteins are classified broadly based on their functions. The proteins involved in morphogenesis are considered structural proteins, while some proteins are responsible for the packaging and release of phage DNA. The proteins that control the lytic cycle of the phage are also involved in the lysis of the host cell and the release of phage particles and thus belong to the host cell lysis module, while some other proteins take part in DNA replication and metabolism of phage particles inside the host cell.

##### 3.8.1.1 Structural proteins

The genomic map of phage MKP-1 broadly consists of 16 ORFs that specifically encode structural proteins. The ordering of these protein-coding genes is mostly congregated in a particular area. Among these ORFs, ORF 22, ORF 25, ORF 26, ORF 27, ORF 28, and ORF 29 encode mainly tail proteins and minor tail proteins. These proteins are involved in the formation of the phage tail system in the Caudovirales family of phages. Minor tail proteins play an important role in assembling the tail subunits. Some of them have enzymatic actions, while some are involved in baseplate formation during phage structure assembly. Three ORF-30, 31, and ORF 32 encode for tail tape measure protein. According to Mahony et al. (2016), this protein controls the length of the phage tail and also helps in the transition of DNA into the host cell cytoplasm during infection. The proteins involved in capsid formation are arranged in an orderly manner with the tail proteins and are encoded by the ORF (38, 39, 41, and 44).

##### 3.8.1.2 Proteins involved in DNA packaging

A bacteriophage genome is closely packed inside the phage capsid, and the packaging of the genome inside the phage capsid is an ATP-driven process. The key proteins that are involved in DNA packaging are the terminase proteins. This is a dimeric protein that consists of large and small subunits, and their role involves the recognition of phage DNA followed by cleavage at specific sites to package the genome inside the capsid. The process of DNA packaging is powered by the ATPase activity of the large subunit of the terminase enzyme (Casjens, 2011). In the phage genome, ORF 47 and ORF 48 encode for the terminase large and small subunits, respectively.

##### 3.8.1.3 Host cell lysis proteins

Two major proteins that drive the phage cell cycle are endolysin and holin. These two proteins are essential for host cell lysis by bacteriophage during their life cycle. Endolysin is generally a muralytic enzyme that degrades the host cell wall; endolysins accumulate in the cytosol fully folded during the phage life cycle (Rahman et al., 2021). On the other hand, Holins are small membrane-bound proteins that accumulate in the membrane and make the membrane permeable to other enzymes such as endolysins. The event is followed by the destruction of the host cell wall, bursting out of the cell, and release of phage particles. It has been reported that holins also control the period of the infective cycle for lytic phages and are subjected to intense pressure to achieve optimal lysis time (Wang et al., 2000). The presence of genes for holin and endolysin enzymes (ORF 4 and ORF 5) ensures the active life cycle of this novel phage.

##### 3.8.1.4 Proteins involved in metabolism and DNA replication

Several other proteins were identified in the MKP-1 genome that might take part in DNA replication and the metabolism system of phage inside the host cell. Enzymes such as primase, exonuclease, and helicase were predicted to be encoded by phage genome, and these proteins are actively involved in DNA replication inside the host cell. Moreover, the presence of DNA-binding protein is also identified in the phage genome that has DNA-binding domains and has a specific or general affinity for single- or double-stranded DNA. The active presence of DNA replication and metabolism-associated proteins in the phage genome would likely reduce the phage dependence on its host bacteria (Peng and Yuan, 2018). Many other proteins were also identified in the phage genome, which we could not annotate but we predict that these proteins are involved mostly in phage metabolism inside host cells.

#### 3.8.2 Genome comparison and phylogeny

Considering the BLASTn analysis, six homologous phages were selected for genome comparison. A comparative table of these homologous phages is shown in Table 2, while the linear arrangement of these phage genomes is presented in Figure 8B. The multiple sequence alignment of phage MKP-1 with the other six relative phages has shown that the genome catalog of these related phages is highly similar and contains specific genes encoding similar structural and functional proteins. The multiple sequence alignment image in Figure 8B shows the presence of conserved domains across the phage genome, which are arranged differently and oriented in opposite directions. To understand the evolutionary relationship, two phylogenetic trees were constructed. The phylogenetic tree, which was constructed based on the whole genome, is presented in Figure 8C and showed the different lineages of phage MKP-1 in the tree. Furthermore, it was clustered with *Klebsiella* phage KPN N141 (NC_047841), *Klebsiella* phage vB_KpnS_15-38_KLPPOU149 (NC_049842), and *Klebsiella* phage KpKT21phil (NC_048143). The other phylogenetic tree, which was constructed based on endolysin protein, is presented in Figure 8D. According to this, the phage MKP-1 is closely related to Klebsiella_phage_KP1801, Klebsiella_phage_MezzoGao, and Klebsiella phage PWKp15.

## 4 Discussion

The rapid emergence of drug-resistant pathogens, especially bacterial species, has created an alarming situation for global public health. The pathogens are opting for new resistance mechanisms, impeding the use of common drugs. According to WHO data, it is estimated that antimicrobial-resistant bacterial infections were directly responsible for 4.95 million deaths globally in 2019 (WHO, n.d.). Therefore, the continuous search for an alternative to regular drugs has shed light on the uses of bacteriophages as treating agents. As, *Klebsiella pneumoniae,* the common intestinal gram-negative bacterial species is one of the leading causes of drug-resistant superbug infections in hospitalized patients, the formulation of effective phage therapy against *Klebsiella* infection is the need of the hour.

In this study, we have investigated a phage, MKP-1, isolated from Mumbai’s Mithi River, known for harboring hospital waste, which potentially harbors clinically resistant bacterial strains. We aim to characterize and evaluate the infectivity of the phage MKP-1 against the bacterium *Klebsiella pneumoniae* so that it could join the phage database as a potential therapeutic candidate against *Klebsiella* infections. Being an encapsulated bacterium, due to the presence of a polysaccharide capsule, *Klebsiella* is protected from external physical stress and thus can show high resistance against antibiotics. However, when the host cells were infected with the phage MKP-1, it was able to decrease the optical density (OD) reading at 600 nm of the host culture from 0.2 to 0.02 (10-folds) within 200 min of infection (Figure 1C), showing its lytic nature. The killing curve also showed that the lytic activity of phage MKP-1 is MOI-dependent, and the higher MOI (MOI 10) was optimal for the greatest reduction of host cells. These findings are quite similar to the previously reported phages such as *Klebsiella* phage ZCKP2 (Fayez et al., 2023), vB_KpnS_Kp13 (Horváth et al., 2020) and P545 (Li et al., 2020). The clear plaques produced on the bacterial lawn also ensure its lytic nature, nullifying it being a temperate phage.

The host range of a phage is one of the key qualities to assess its usefulness in phage therapy. The greater the host range within a particular species, the more effective a particular phage will be against the target pathogen causing a specific disease (Ranveer et al., 2024) However, a phage should not have any killing effects on other bacterial species so as not to affect the friendly commensal bacteria. In this regard, the host range of phage MKP-1 attributed to its usefulness as it showed its lytic activity against three strains of *Klebsiella* but not against any other species.

Considering the growth curve of this phage, a high latent period (40 min) of the phage is often an indicator of high burst size (Abedon et al., 2001), confirming its effective use as therapeutics. The large burst size (more than 200 pfu per cell) is comparable to other *Klebsiella* phages such as phage KP 1801 and ZCKP2 (Wintachai et al., 2020; Fayez et al., 2023). However, unlike phage MKP-1, other Siphoviridae *Klebsiella* phages have been shown to have a latent period of approximately 20 min (Horváth et al., 2020; Fayez et al., 2023). Some of the previous studies have shown that the latent period of a phage life cycle could vary based on host cell density and could impact phage therapy protocols significantly (Abedon et al., 2001).

For a successful infection, phage particles have to adsorb effectively onto the host cell membrane by receptor-mediated binding. Based on the adsorption kinetics, we could say that this phage also obeys the biphasic growth kinetics [3(B)] like most other phages (Chatterjee and Maity, 1984; Lamiaa et al., 2023), as the adsorption rate of this phage was faster during the initial phase of infection and gradually decreased with time.

Phage sensitivity against environmental factors such as temperature, pH, and salinity is a tricky issue for successful phage therapy. Studies have suggested that temperature could effectively reduce the rate of phage infection (Olson et al., 2004), whereas the pH can affect the stability of the phage. According to our findings, the persistent stability of this phage under a broad range of temperatures (10°C–60°C), pH (3–12), and salinity (up to 600 mM) is similar to other reported *Klebsiella* phages (Horváth et al., 2020; Li et al., 2020; Fayez et al., 2023) and could contribute significantly to assess its utility. Phage MKP-1 could be considered a thermotolerant phage and is also quite resistant under pH stress. However, like most other microbes, UV showed high inactivating effects as approximately 10 s of UV exposure was able to decrease phage survival rate to nearly 25% (Figure 4).

The structural details of host–pathogen interaction showed the same pattern of phage growth curve (Figure 3A), having a comparatively long latent period. Intact phages were attached to the host after 5 min of infection (Figure 5A), but complete lysis was observed after 40 min of infection. The ultrathin sections of the *K. pneumoniae* infected with the phage MKP-1 have represented the structural insight of host–pathogen interaction. When phages are yet to release their DNA, the cellular membrane of the host is intact and all the cellular materials are visible. After 15 min of incubation, when the phages have transferred their DNA to the host, the host cell membrane is disrupted at the point of contact, and the cells started to get disorganized inside. However, after approximately 40 min of incubation with the phage, the host cells were observed to be completely disorganized, and all the cellular material leaked out from the cell satisfying the growth curve obtained for the phage MKP-1 (Figures 5C–E).

One of the major reasons for the virulence of *K. pneumoniae* is their aptness to form biofilms, the complex bacterial communities that may form by involving one or more species (Balestrino et al., 2005). The formation of biofilms induces increased resistance to external stress, including antimicrobial substances (Brindhadevi et al., 2020; Wang et al., 2020). Thus, we have assessed the lytic activity of phage MKP-1 in reducing biofilms formed on glass coverslips and 96-well plates. Phage MKP-1 was active against the host in both cases and successfully reduced bacterial population in biofilms significantly. We also wanted to check the ideal incubation time of phage with host biofilm for optimal lysis and concluded that 12 h of incubation with the host cell biofilm is optimum rather than 18–24 h. In some common human diseases such as cystic fibrosis, urinary tract infections, pneumonia, and inflammatory bowel disease, biofilm formation often takes place due to polymicrobial infections involving two or more bacterial species (Short et al., 2014). This phenomenon is often accountable for fewer effects of antimicrobial therapies (Short et al., 2014; de Vos et al., 2017). Therefore, when the biofilm degradation ability of phage MKP-1 was tested against mixed infection of *Pseudomonas aeruginosa and K. pneumoniae*, our findings suggested that this phage affected the mixed biofilm but not very significantly (Supplementary Figures S3, S4).

The detailed genomic analysis of the phage genome revealed useful information that could place it in the pools of potential bacteriophages. The phage genome codes for most of the essential proteins that make it independent and stable while infecting the host. Its genome does not possess any unwanted genes such as genes for antibiotic resistance, lysogeny, or any toxins. This phage MKP-1 showed similar genomic organization to other *Klebsiella* phages such as *Klebsiella* phage mtp25, KPN N141, mtp30, phage KL, vB_Kpn_K2069PH1, and phage KpKT21phi1. The multiple sequence alignment with these phage genomes showed the presence of specific conserved domains across the phage genome. However, the genes for these domains are differently arranged in phage MKP-1 to other phages. This could be due to orientation and inversion of gene locus within the phage genome (Figure 8B). In addition, the gene lengths for various conserved domains vary in different phages. Based on the whole genome sequences and the endolysin protein, the phylogenetic analysis confirms its different lineage and clustered it with *Klebsiella* phages KPN N141, KpKT21phil. As it is clustered together with T-5-like *Klebsiella* phages, it can be considered a new species of T-5-like *Klebsiella* phages.

## Data availability statement

The datasets presented in this study can be found in online repositories. The names of the repository/repositories and accession number(s) can be found in the article/Supplementary material. The complete genome of Klebsiella phage MKP-1 is deposited in NCBI GenBank with Accession number PP627509.

## Author contributions

SD: Conceptualization, Data curation, Formal analysis, Investigation, Methodology, Software, Validation, Writing – original draft. SK: Funding acquisition, Supervision, Writing – review & editing.
