# Supplementary material for: Physiochemical characterization of a potential Klebsiella phage MKP-1 and analysis of its application in reducing biofilm formation
Source: Front Microbiol. 2024 Jul 17;15:1397447. doi: 10.3389/fmicb.2024.1397447 (PMC11288805; doi:10.3389/fmicb.2024.1397447)
Supplement: Supplementary file 1 [file Table_1.docx]

**Supplementary Table 1: List of proteins encoded by the MKP-1 phage genome with their proposed function.**

| **Predicted Proteins** | **Proposed function** |
| --- | --- |
| **Rz-like spanin** | Host interaction |
| **Endolysin** | Host lysis |
| **Holin** | Host lysis |
| **Deoxynucleoside monophosphate kinase** | Metabolic protein |
| **Polynucleotide kinase** | Metabolic protein |
| **DNA repair exonuclease** | DNA replication |
| **DNA methyltransferase** | DNA replication |
| **Transcriptional regulator** | Metabolic protein |
| **DNA primase** | DNA replication |
| **Exonuclease VIII** | Host interaction |
| **Erf-like ssDNA annealing protein** | DNA replication |
| **Single strand DNA binding protein** | DNA replication |
| **Tail fiber protein** | Structural protein |
| **Membrane-associated protein** | Receptor protein |
| **Outer membrane protein** | Receptor protein |
| **Tail protein** | Structural protein |
| **Minor tail protein** | Structural protein |
| **Tail length tape measure protein** | Structural protein |
| **Tail terminator** | Structural protein |
| **Major tail protein** | Structural protein |
| **Head closure Hc1** | DNA packaging |
| **Virion structural protein** | Structural protein |
| **Major head protein** | Structural protein |
| **Head maturation protease** | Structural protein |
| **Head morphogenesis** | Structural protein |
| **Terminase large subunit** | DNA translocation |
| **Terminase small subunit** | DNA translocation |
| **Membrane protein** | Receptor protein |
| **DNA helicase** | DNA replication |
