# Supplementary figures and images for: Physiochemical characterization of a potential Klebsiella phage MKP-1 and analysis of its application in reducing biofilm formation
Source: Front Microbiol. 2024 Jul 17;15:1397447. doi: 10.3389/fmicb.2024.1397447 (PMC11288805; doi:10.3389/fmicb.2024.1397447)

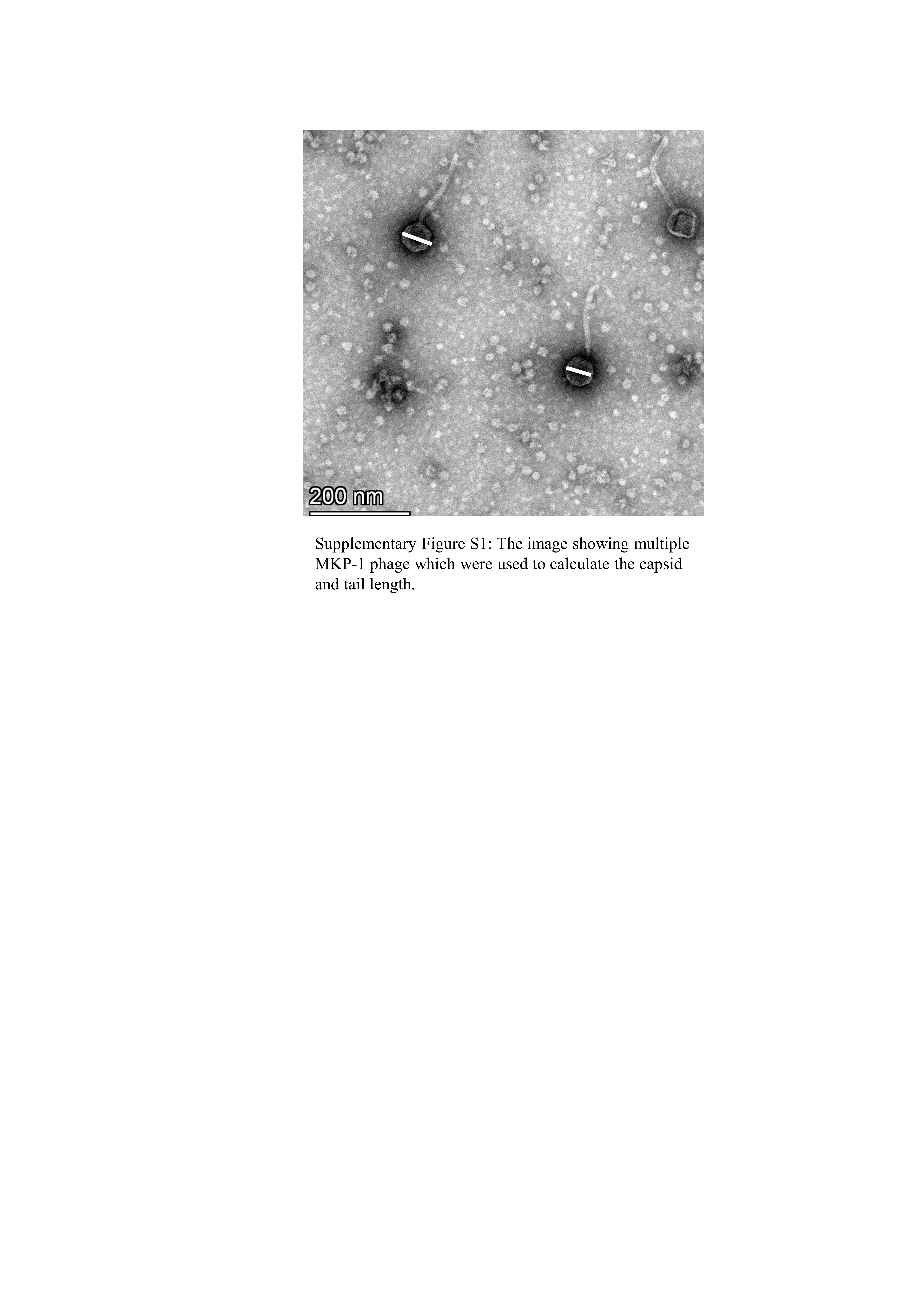

Supplement: Supplementary file 2 [file Image_1.TIF]

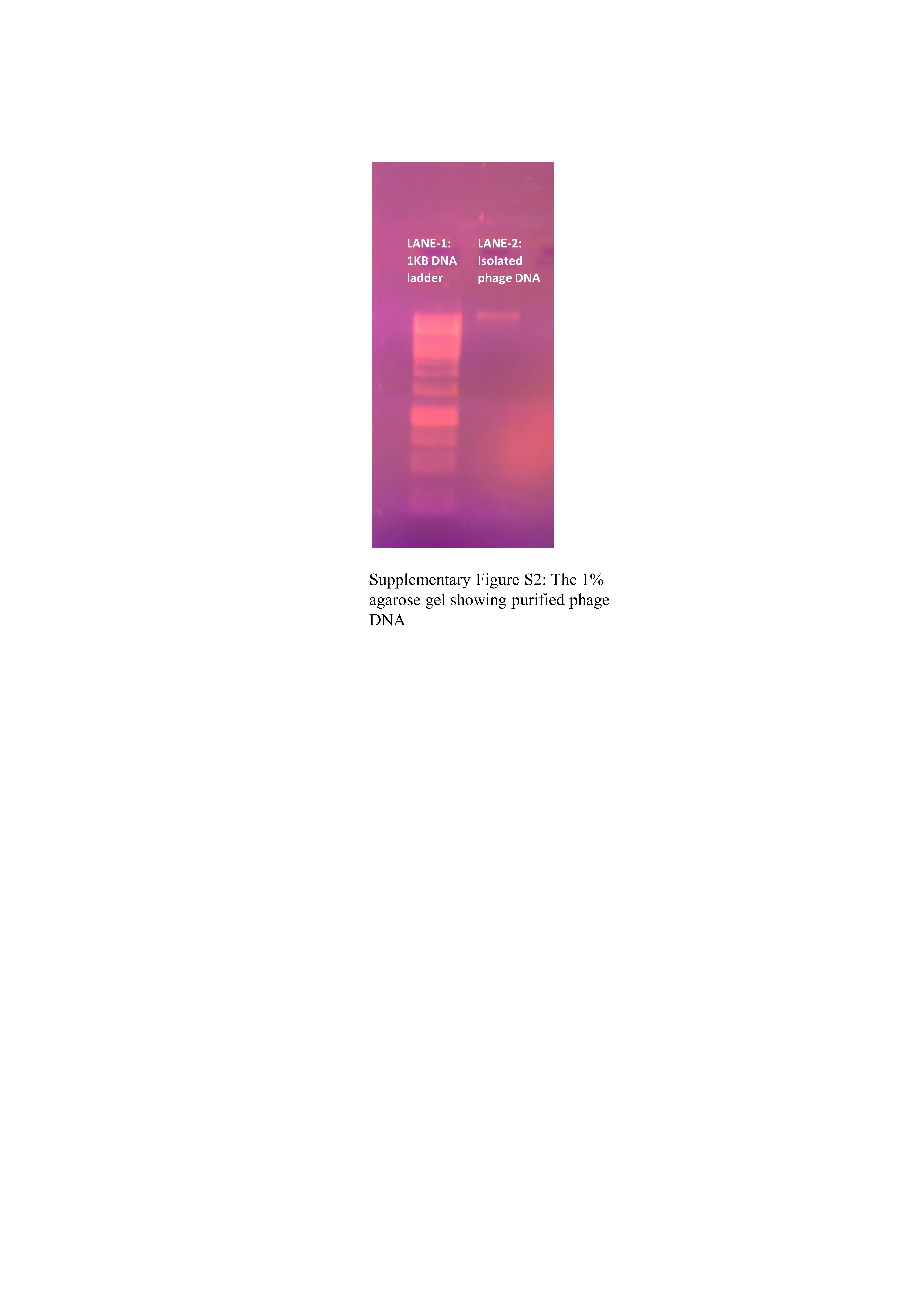

Supplement: Supplementary file 3 [file Image_2.TIF]

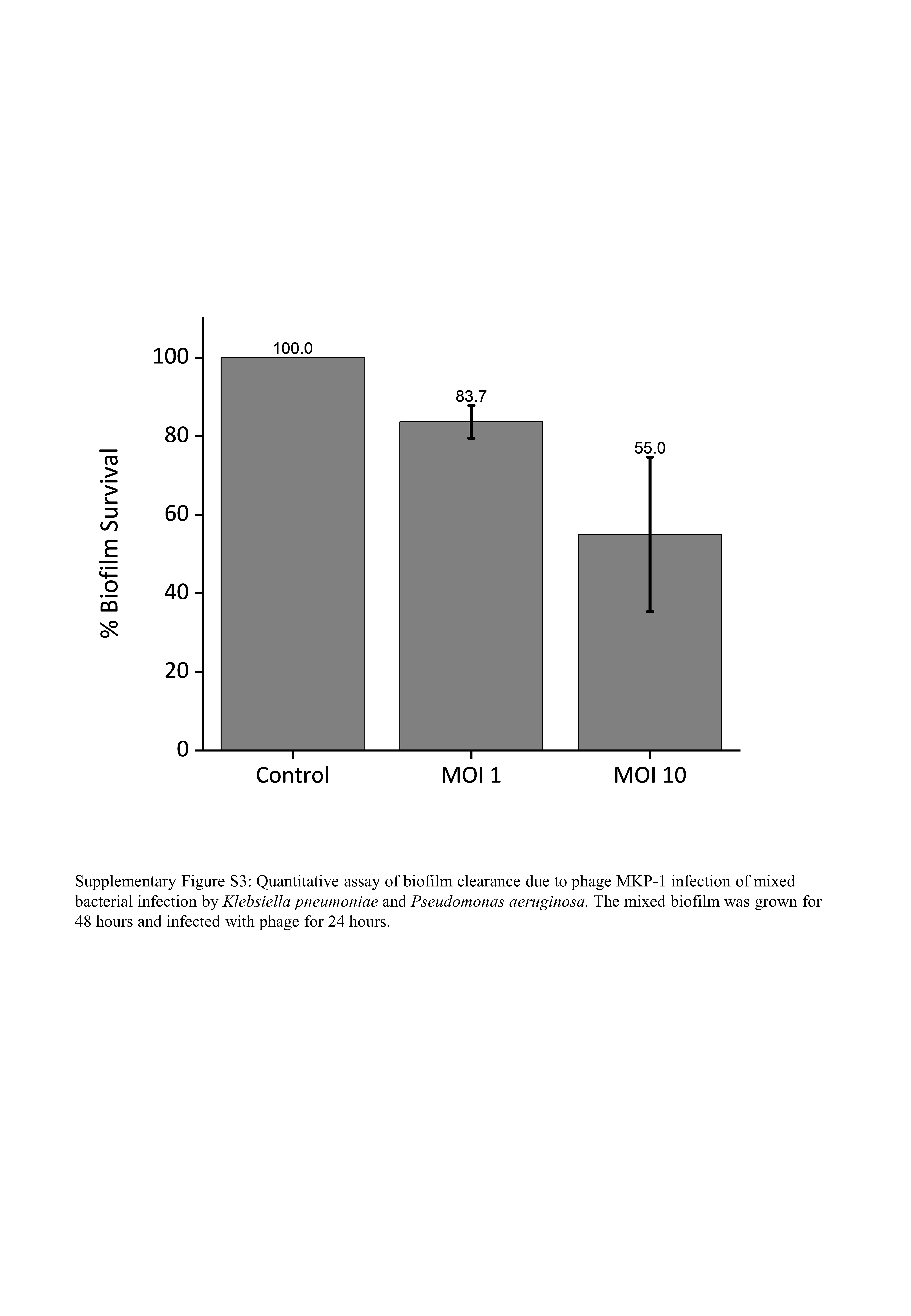

Supplement: Supplementary file 4 [file Image_3.TIF]

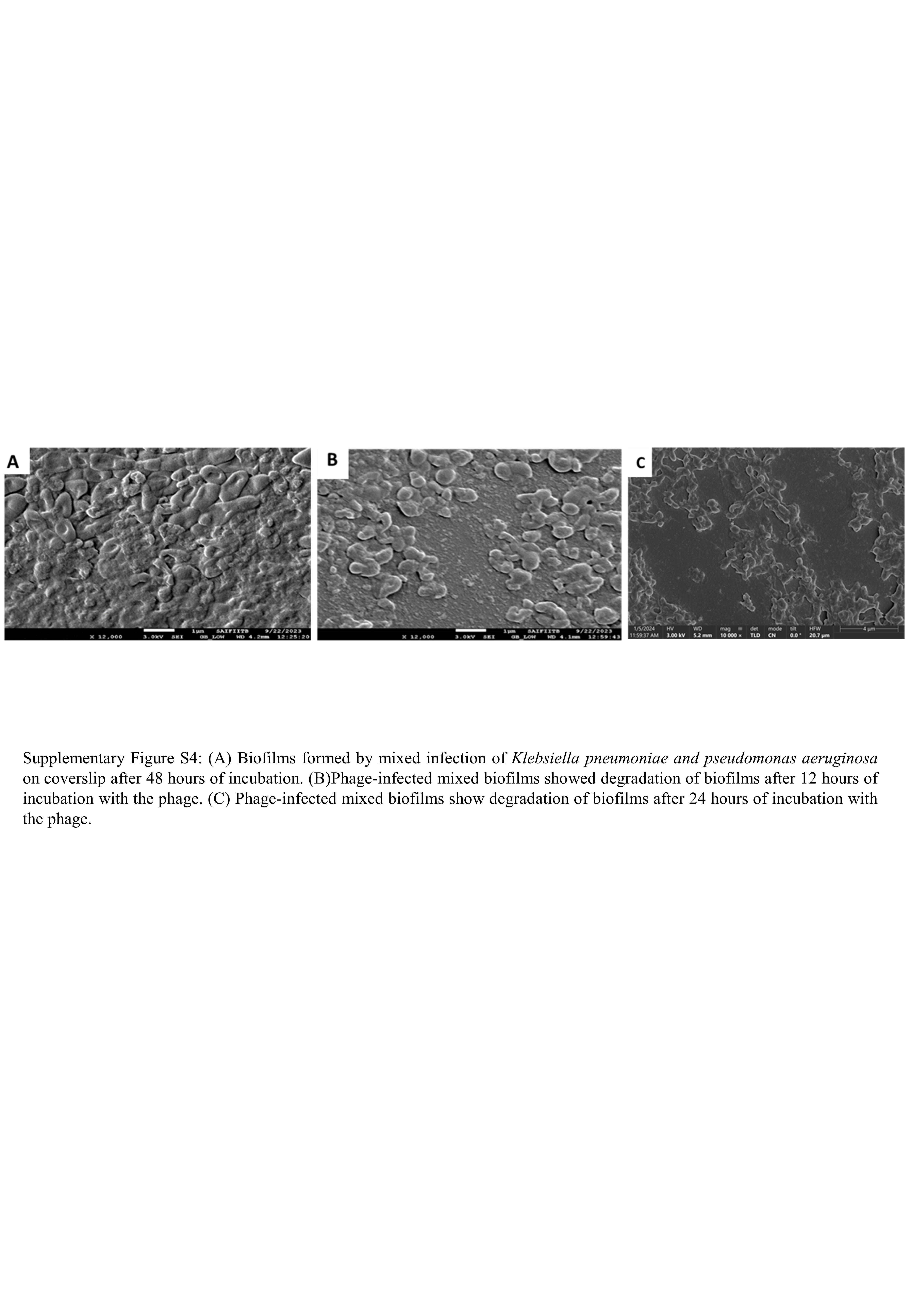

Supplement: Supplementary file 5 [file Image_4.TIF]

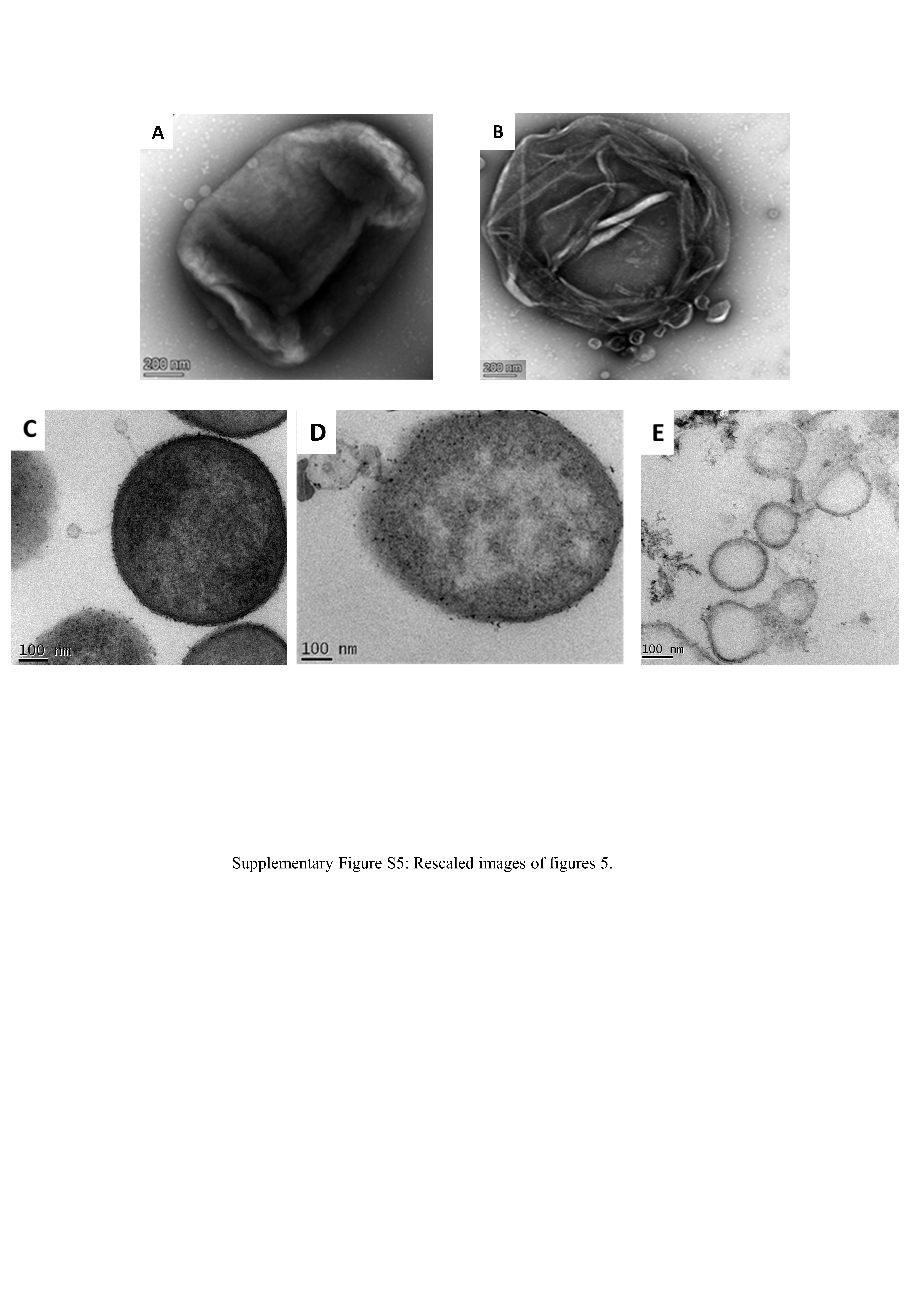

Supplement: Supplementary file 6 [file Image_5.TIF]
